# BK Polyomavirus Infection in Kidney Transplantation: A Comprehensive Review of Current Challenges and Future Directions

**DOI:** 10.3390/ijms252312801

**Published:** 2024-11-28

**Authors:** Nicole Nourie, Céline Boueri, Hoang Tran Minh, Gillian Divard, Carmen Lefaucheur, Maud Salmona, Simon B. Gressens, Kevin Louis

**Affiliations:** 1 Kidney Transplant Department, Saint Louis Hospital, Assistance Publique-Hôpitaux de Paris, Université Paris Cité, 75010 Paris, France; 2 Human Immunology and Immunopathology, Inserm UMR 976, Université Paris Cité, 75010 Paris, France; 3 Laboratory of Virology, Saint Louis Hospital, Assistance Publique-Hôpitaux de Paris, Université Paris Cité, 75010 Paris, France; 4 Department of Infectious Diseases, Saint Louis Hospital, Assistance Publique-Hôpitaux de Paris, Université Paris Cité, 75010 Paris, France; 5 Team 3I Brain, Inserm UMR 1141, 75019 Paris, France

**Keywords:** kidney transplantation, BK polyomavirus infection, biomarkers

## Abstract

BK polyomavirus (BKPyV) infection of the kidney graft remains a major clinical issue in the field of organ transplantation. Risk factors for BKPyV-associated nephropathy (BKPyVAN) and molecular tools for determining viral DNA loads are now better defined. BKPyV DNAemia in plasma, in particular, plays a central role in diagnosing active infection and managing treatment decisions. However, significant gaps remain in the development of reliable biomarkers that can anticipate BKPyV viremia and predict disease outcomes. Biomarkers under active investigation include urine-based viral load assays, viral antigen detection, and immune responses against BKPyV, which may offer more precise methods for monitoring disease progression. In addition, treatment of BKPyVAN is currently based on immunosuppression minimization, while the role of adjunctive therapies remains an area of active research, highlighting the need for more personalized treatment regimens. Ongoing clinical trials are also exploring the efficacy of T-cell-based immunotherapies. The clinical management of BKPyV infection, based on proactive virological monitoring, immune response assessment, integrated histopathology, and timely immunosuppression reduction, is likely to reduce the burden of disease and improve outcomes in kidney transplantation.

## 1. Introduction

BK polyomavirus (BKPyV) was first discovered in 1971 and named after the initials of the patient from whom it was isolated and who presented ureteral stricture 3 months after kidney transplantation [1]. It was not until 1993 that the first biopsy-proven case of BKPyV-associated nephropathy (BKPyVAN) was documented [2]. BKPyV is a ubiquitous and widespread double-stranded DNA virus of the *Polyomaviridae* family, to which 85–90% of the general adult population is exposed, that causes an asymptomatic primary infection that commonly occurs during childhood, as shown by serological studies performed in humans [3]. BKPyV is thought to be transmitted through the digestive or respiratory tracts, but its precise mechanisms of infection remain poorly understood. Following initial exposure, BKPyV remains quiescent in the epithelial cells of the renal medulla and uroepithelial cells of the urinary tract. However, low-level viral persistence can be disrupted when immunosuppressive drugs weaken immune defenses following kidney transplantation, increasing the risk of viral reactivation. Viruria is detected in up to 30% of transplanted patients, half of whom will develop BK viremia, and almost half of viremic patients will progress to BKPyVAN. In total, 5–10% of kidney transplanted patients will develop BKPyVAN [4,5]. In previous decades, BKPyVAN represented one of the most important causes of kidney graft dysfunction with reports describing 60–80% of cases resulting in graft loss after plasma viral replication. Since then, advances in diagnostics, understanding of underlying BKPyV pathogenicity and antiviral immune responses, and reducing immunosuppressive drug pressure have helped to slightly improve clinical outcomes, thereby reducing the incidence of kidney graft loss [6,7].

The onset of BKPyV replication and infection depends on several donor-related risk factors such as deceased donor transplant, BKPyV genotype, and genotype mismatch with the recipient, as well as urinary BKPyV shedding. The recipient risk factors for BKPyVAN include older age, male sex, BKPyV-seronegative antibody status, and previous kidney transplantation [8]. Some HLA genotypes and killer cell immunoglobulin-like receptors (KIR) are also considered protective or predisposing to BKPyV infection as they may modulate natural killer cell antiviral response [9,10]. However, the most important risk factor remains transplantation-associated factors, particularly immunosuppressive regimens with the use of tacrolimus (versus the use of cyclosporine) and a higher dose of corticosteroids and anti-T-cell lymphocyte globulin but also the use of ureteric stents [11]. Therefore, there is a higher risk of BKPyV replication after desensitization protocols and immunosuppressive treatment intensification following a rejection episode [12]. The spectrum of BKPyV-related diseases can now be categorized within a framework recently updated by international consensus guidelines [13], aimed at pragmatically guiding patient management and future research. This framework includes several diagnostic categories: “possible BKPyVAN” characterized by BK viruria exceeding 10 million copies/mL, the presence of decoy cells, or BK virions identified via electron microscopy but with undetectable BK viremia; “probable BKPyVAN” defined by a BK viremia level greater than 1000 copies/mL sustained for over two weeks; “presumptive BKPyVAN” indicated by BK viremia exceeding 10,000 copies/mL; and “biopsy-proven BKPyVAN”, which requires evidence of cytopathic effects plus immunohistochemistry and a specific diagnostic test identifying BKPyV as opposed to JC polyomavirus.

In the absence of prophylactic or therapeutic treatments, current management strategies depend on tapering immunosuppressive regimens when patients experience viral reactivation or graft damage. To mitigate the risk of allograft immune rejection associated with this approach, detection of viral reactivation is crucial, particularly in the early post-kidney-transplant period. Screening protocols are currently based on the quantification of plasma BKPyV DNA using PCR. The International Consensus 2024 guidelines now recommend systematic monthly screening of BKPyV DNA in kidney transplant recipients until the ninth month post transplantation, followed by screenings every 3 months until the second year (or third year for pediatric patients). In addition, measuring plasmatic BKPyV DNAemia should be conducted in clinical contexts suggestive of complications, such as deterioration in kidney function. This monitoring strategy faces multiple limitations. First, the detection of BKPyV-DNAemia likely indicates the high replication state of the virus, as it is often preceded by significant BKPyV-DNA loads in the recipient’s urine. However, monitoring viral loads in urine is limited by the lower positive predictive value for BK nephropathy compared to plasma BKPyV-DNAemia, as well as the variability introduced by physiological changes in urine composition, which can complicate its interpretation [13]. Additionally, graft dysfunction, typically assessed through plasma creatinine levels, is an unreliable indicator of graft damage since such damage can occur without elevated creatinine levels [14]. This underscores the continuum of virus-induced injury, which can progress to irreversible fibrotic lesions and graft dysfunction. Therefore, more sensitive and early markers of viral replication and control are needed to adjust management strategies promptly.

In light of the challenges in diagnosing and treating BKPyVAN, this review outlines recent advancements in the search for effective biomarkers for timely and precise diagnosis. It also examines current therapeutic options and ongoing clinical trials aimed at enhancing preventive and curative strategies for BKPyV-infected kidney transplant recipients.

## 2. Biomarkers

BKPyV DNAemia in plasma is a reproducible diagnostic tool for BKPyVAN but lacks sensitivity for the early detection of kidney graft damage. Thus, novel assessments of disease stage and progression based on the characterization of immune responses to BKPyV (BKPyV-specific cell-mediated immunity, BKPyV-specific antibodies, and urinary chemokines), and based on the evaluation of kidney tissue damage (polyomavirus–Haufen test, gene expression in graft biopsy, and urine) are under active investigation. On one hand, more sensitive assays, such as the PyV-Haufen test, donor-derived cell-free DNA, and urinary chemokine profiles, are being scrutinized for their ability to detect graft damage early. On the other hand, biomarkers that predict the effectiveness and reconstitution of the immune response—encompassing adaptive cellular and humoral responses, as well as antiviral innate responses—are being explored to guide immunosuppression management. These markers of antiviral immune response and graft injury may represent additive tools for early and precision diagnosis in BKPyVAN and may represent surrogate markers of response to therapy and the prediction of late graft function.

### 2.1. Urinary Polyomavirus–Haufen Test

In 2009, electron microscopy (EM) unveiled intriguing three-dimensional clusters in urine samples from patients with BKPyVAN. These clusters, consisting of at least six polyomaviruses, were named “Haufen”, a German term that literally translates to pile, or in this case accumulation or clustering. Utilizing immunohistochemical techniques to detect polyomavirus capsid proteins, coupled with immunogold labeling EM employing a mouse monoclonal anti-human Tamm–Horsfall antibody in kidney biopsies, researchers were able to unravel the cascade of events precipitating from virion multiplication within tubular cells, triggering epithelial cell injury and lysis. Subsequently, the release of these virions into the tubular lumen culminated in the formation of these viral aggregates, eventually detectable in urine [15]. In a cohort of 809 transplant recipients, the urinary polyomavirus–Haufen (PyV-Haufen) test emerged with high sensitivity (100%) and specificity (98%) compared to alternative assays targeting viral replication markers such as urinary decoy cell shedding, BKPy-DNAemia, and DNAuria. It also exhibited a strong correlation with disease severity and the Banff classification. Moreover, the gradual decline in PyV-Haufen levels mirrored the resolution of nephropathy, aligning with diminishing Banff pvl scores. However, despite its diagnostic value, practical implementation poses challenges owing to its reliance on EM. Therefore, the test finds its place not in routine screening but rather as a complementary tool for patients exhibiting viral replication evidence on primary testing, in order to determine the presence of PyVN in these patients, particularly within the high-risk subgroup susceptible to biopsy complications [16]. Furthermore, this non-invasive assay emerges as a specific marker for kidney injury attributable to viral replication. This was corroborated by findings from a uromodulin knockout mice model, where none of the uromodulin knock-out mice exhibiting signs of PyVN displayed urinary PyV–Haufen shedding, in contrast to 71% of wild-type mice [17].

### 2.2. Urinary Chemokines

Chemokines are pivotal in regulating leukocyte recruitment and migration, crucial not only in viral infections but also in inflammatory responses and tissue injury. Among them, C-X-C Motif Chemokine Ligand (CXCL)10 binds to CXCR3, expressed on various immune cells, including Th1 and monocytes, which secrete interferon-gamma (IFN-γ) which in turn augments CXCL10 production [18,19]. Human tubular epithelial cells stimulated with IFN-γ and TNF-α can also secrete CXCL9 and CXCL10 [20]. While these chemokines are implicated in immune responses against BKPyV, their potential diagnostic value for BKPyVAN is being investigated. Increased urinary CXCL9 and CXCL10 have been long reported during BKPyVAN with high sensitivity but lower specificity since they can reflect graft inflammation induced by BKPyV or caused by other etiologies [21]. In a recent retrospective study by Mayer, K.A. et al., 19 cases of biopsy-proven BKPyVAN selected from the Vienna transplant cohort were compared to patients with known T-cell-mediated rejection, antibody-mediated rejection, and stable grafts. Patients with BKPyVAN exhibited elevated levels of urinary CXCL10 and CXCL9 compared to those with antibody-mediated rejection, but no significant difference was observed when compared to patients with T-cell-mediated rejection [22]. However, in a retrospective study by Weseslindtner L. et al., 56 patients who exhibited isolated BKPyV replication, evidenced by at least one positive DNAemia result (DNA viral loads > 1000 copies/mL), were observed to have rising urine CXCL10 levels in parallel to disease progression, from BK DNAuria to DNAemia and histological evidence of BKPyVAN. This increase correlated with heightened viral replication, the excretion of decoy cells, and a decline in kidney function. Additionally, blood levels of CXCL10 and also CCL8 were notably higher in patients with elevated levels of viral replication, decoy cell shedding, and a decline in glomerular filtration rate (GFR) [23]. Regardless of this lack of specificity, urine CXCL10 shows prognostic potential when compared to other testing such as blood and urine BKPyV load as well as histology. In a longitudinal study of 60 patients with BKPyV-DNAemia, the urine CXCL10/creatinine ratio emerged as the sole predictor of graft function decline. The authors were able to distinguish between high- and low-risk patients for graft function decline using a urine CXCL10/creatinine threshold of 12.86 ng/mmol. In viremic patients, urine CXCL10 levels at the time of biopsy were associated with graft functional decline, independent of baseline eGFR, blood viral load, or diagnosis of BKPyVAN. While urinary CXCL10 levels correlated with BKPyV DNAemia trends, they did not show a correlation with BKPyV viruria. Moreover, the time-adjusted urine CXCL10/creatinine AUC was notably higher in patients who later experienced rejection, highlighting the inflammatory response during BKPyV infection preceding rejection, and this was regardless of immunosuppressive regimen adjustments, as shown by Tinel C. et al. [24]. Recently, urinary CXCL10 as a diagnostic marker was revisited in a secondary analysis of a randomized trial, by Haller J. et al., involving 241 kidney transplant recipients. Urinary CXCL10 was assessed at seven different screening intervals and at clinically relevant times. A urinary CXCL10 level of ≤3 ng/mmol was found to effectively exclude the shedding of ≥3 decoy cells with a negative predictive value (NPV) of 97%, along with BKPyV DNAemia with an NPV of 99%, and was associated with a reduced risk of rejection. However, urine CXCL10 alone may not serve as a definitive diagnostic tool for BKPyV infection or differentiate it from graft rejection. Nonetheless, urine CXCL10 could serve as an additional tool, along with BKPyV DNAemia testing, to promptly exclude an ongoing inflammatory graft process and try to distinguish graft rejection from BKPyVAN [25].

### 2.3. Donor-Derived Cell-Free DNA

Donor-derived cell-free DNA (dd-cfDNA) is a valuable non-invasive tool that can reflect ongoing graft injury with a high NPV, which may help avoid invasive biopsies in high-risk patients. Elevated plasma dd-cfDNA levels have been associated with rejection episodes and BKPyVAN [26]. Early studies examining the correlation between dd-cfDNA and BKPyVAN primarily focused on urinary fluctuations, given that dd-cfDNA shedding likely reflects tubular epithelial cell infection. This was examined in a single-center study including 93 BKPyV-infected patients, with cases classified into categories based on the American Society of Transplantation guidelines (proven, probable, possible, and resolving BKPyVAN). Urine dd-cfDNA significantly outperformed BKPyV DNAemia in distinguishing BKPyVAN from other diagnoses (area under the curve (AUC): 0.842 vs. 0.660). Furthermore, among patients with stable serum creatinine at BKPyVAN diagnosis, a high proportion had elevated urinary dd-cfDNA levels, detecting graft injury before creatinine elevation, thus prompting timely intervention. Additionally, the subsequent decline in urine dd-cfDNA in BKPyVAN patients after onset suggests its potential for assessing therapeutic efficacy [27]. To further elaborate on the use of urinary dd-cfDNA in differentiating between BKPyVAN and rejection, another study enrolled 60 patients categorized as 12 with stable graft function, 22 with T-cell mediated rejection, 21 with proven BKPyVAN, and 5 patients with possible BKPyVAN. Remarkably, using a cut-off value of 7.81 ng/mL, urinary dd-cfDNA concentrations successfully distinguished proven BKPyVAN from TCMR of grade I, with an AUC of 0.848 [28]. Furthermore, this result was also supported in another study, which identified a threshold of 6.08 ng/mL [29]. On the other hand, dd-cfDNA measured in plasma failed to distinguish between TCMR and BKPyVAN [21]. But when combined with clinical features, to develop a regression model, programmed monitoring of changes in Δdd-cfDNA (obtained over time by subtracting the baseline dd-cfDNA from dd-cfDNA at subsequent time points) was shown to be useful in diagnosing BKPyVAN versus other types of graft injury [30].

### 2.4. BK-Specific Cell-Mediated Immunity

BKPyV-specific cell-mediated immunity (CMI) assays measure a variety of differentiation, activation markers, and effector functions such as cell proliferation, cytotoxicity, and cytokine production in response to BKPyV. Proportions of blood BKPyV-specific T cells are detected at very low levels in healthy humans but also in patients with active BKPyV replication [31]. IFN-γ is a common functional read-out for CMI, and BKPyV-specific enzyme-linked immune absorbent spot (ELISPOT) assays have been developed to detect BKPyV-specific T cells with greater sensitivity [32,33]. A recent meta-analysis by Udomkarnjananun, S. et al. investigated the diagnostic performance of the BKPyV-specific IFN-γ ELISPOT, dividing patients based on positive and negative ELISPOT results. Nine articles were included, utilizing various BKPyV viral antigens (large T antigen, small t antigen, VP1, VP2, VP3, or mixed antigens) and differing definitions of BKPyV infection (positive decoy cells, viruria, viremia, or BKPyVAN). Patients with a negative ELISPOT result were at increased risk of active BKPyV replication. The pooled sensitivity was high at 0.95, with a pooled specificity of 0.88. The mean number of IFN-γ producing cells was significantly lower in patients with active BKPyV infection compared to those with resolving BKPyV infection [34]. In 2023, another study by Bae, H. et al. showed that pre-transplant BKPyV-ELISPOT results were lower in patients who developed BK viremia post transplant than those without any BK viremia. Sensitivity was 71.4% and specificity was 54.4% for the prediction of post-transplant BK viremia using BKPyV-ELISPOT with cut-off ≤ 53 spots/3 × 10^5^ cells. Additionally, a combination of high donor BKPyV-IgG, low recipient BKPyV-IgG, and low BKPyV-ELISPOT was associated with improved specificity of 91.1% to predict later BK viremia [35]. Although a number of BKPyV-specific T cells in peripheral blood can be very low, several studies were able to detect changes in phenotypes in CD8 T cell compartments during BKPyV infection. CD28-negative effector memory and terminally differentiated effector memory CD8 T cell phenotypes were associated with the clearance of BK-DNAemia, while effector-memory differentiation was impaired in patients with high viral load and BKPyVAN [36]. In a study by Ahlenstiel-Grunow, T. et al., levels of BKPyV-specific CD4 and CD8 T cells were negatively correlated with the subsequent duration of BKPyV DNAemia, and after reducing immunosuppressive therapy, levels of BKPyV-specific CD4 T cells increased while those of plasma BK DNAemia declined [37]. When assessed longitudinally, BKPyV-specific CD8 T cell responses to pools of immunodominant 9mers (9mer peptide pool) from the early viral gene region at 6 months and 12 months post transplant were associated with the clearance of viremia [38]. Thus, CMI assays may represent biomarkers as a complement of BKPyV DNAemia for guiding immunosuppression reduction and for BKPyV risk assessment.

### 2.5. BKPyV-Specific Antibodies

BKPyV-specific antibodies can be assessed using a solid phase assay against viral proteins, most commonly VP1. Detection of BKPyV-specific antibodies by VP1 ELISA in kidney donors has been associated with an increased risk of BKPyV DNAemia and BKPyVAN in kidney recipients, particularly when donor IgG levels were high or when they were undetectable or low in the kidney recipient [39]. Pairing of high BKPyV-seroreactive donors with low-seroreactivity recipients resulted in a 10-fold increased risk of BK viremia. While recipient BKPyV seroreactivity was not significant, donor BKPyV-seroreactivity was a strong pre-transplant factor associated with viremia and BKPyVAN [40]. This supports the hypothesis that donor BKPyV-seroreactivity potentially reflects the BKPyV infectious load of the kidney graft. Other studies have confirmed that donor IgG anti-BKPyV levels determined using ELISA have been shown to significantly correlate with BKPyV replication in DNAemia, the appearance of IgM, and BKPyVAN [41]. However, IgG anti-BKPyV levels did not correlate with the clearance of BKPyV viremia in kidney recipients [38]. Interestingly, kidney recipients with high BKPyV genotype-specific neutralizing antibody (Nab) levels against the replicating strain had a lower risk of developing BKPyV DNAemia [42]. In this study by Solis M. et al., BK genotype mismatch between recipient neutralization profiles at transplant and their subsequently replicating strain significantly increased the risk of developing BKPyV DNAemia. A low NAb titer against the donor strain at transplant is significantly associated with BKPyV DNAemia after transplant. A genotype-specific NAb titer may represent a biomarker for risk stratification at the time of transplant.

### 2.6. Gene Expression in Graft Biopsy and Urine

Since a kidney graft biopsy may be falsely negative due to sampling errors, suboptimal sensitivity of SV40 immunohistochemistry, and a lack of medullar portion, there is a need for a new tool to facilitate the assessment of BKPyVAN diagnosis in tissue. A recent study by Adam, B.A. et al. used a Nanostring gene expression panel to assess tissue with BKPyVAN versus other confounding clinical scenarios [43]. The authors identified five genes (VP1, VP2, VP3, Agnoprotein, and LTAg) on native kidney tissues with BKPyVAN and showed that these genes were more sensitive for identifying BKPyV-positive cases in kidney transplant biopsies when added to histology than histology alone. Interestingly, the polyomavirus five-gene set was able to distinguish BKPyVAN from T-cell mediated rejection which can display similar histopathology lesions. However, the five-gene set was not able to predict graft failure and measure response to BKPyVAN therapy. Another study showed that *RBBP7* (retinoblastoma binding protein 7), a transcript that affects DNA replication and controls cell proliferation, may distinguish BKPyVAN from other diagnoses and was associated with specific immune cell infiltration in BKPyVAN [44]. Urinary VP1 mRNA was also shown to be able to distinguish BKPyVAN from other diagnoses with a sensitivity of 86% and a specificity of 100% [45]. Standardized methods for graft and urine BKPyV-specific transcripts are needed before implementation in clinical practice. Also, validation of these transcriptional signatures using in vitro models and immune cell stimulation would be useful.

## 3. Adjuvant Therapies for BKPyV-Associated Infection

To date, the validated management strategy relies on reducing immunosuppression, aiming for directed immunological control. This approach may appear straightforward but is practically complex, challenging to standardize, and predisposed to rejection episodes. BKPyV-DNAemia is dynamic and inversely correlated with an efficient antiviral immune response. Thus, clinical recommendations now support stepwise immunosuppression minimization with a reduction in the dose of the antimetabolite by at least 50%; then, if BKPyV DNAemia does not decrease, discontinuation of the antimetabolite plus tapering of the corticosteroid dose to 5–10 mg/d of prednisone or equivalent is initiated [13]. Another option is to reduce the first dose of the calcineurin inhibitor by 25–50% before reducing the antimetabolite dose by 50%. However, target concentrations for dose reduction of calcineurin inhibitors are not well defined and need to be individualized. Although there is no specific antiviral treatment against BKPyV, several studies have evaluated the potential additive value of diverse adjuvant therapies to treat BKPyVAN in addition to stepwise IS minimization. These data are summarized in Table 1 and are mostly based on single-center, retrospective transplant center experience and/or non-controlled case series, resulting in insufficient strength to date to support evidence of clinical efficacy.

### 3.1. Leflunomide

Inhibition of the intracellular protein kinase pathways activated by BKPyV represents one of the therapeutic strategies for BKPyVAN. Leflunomide is an anti-metabolite approved for the treatment of rheumatoid arthritis which inhibits pyrimidine synthesis, resulting in anti-proliferative and anti-inflammatory effects. Its metabolite, teriflunomide (A771726) has been found to inhibit the replication of BKPyV in vitro and in animal models [46,47]. Although leflunomide’s mechanism of action in decreasing BKPyV replication is not fully understood, it is thought to be similar to its effects on CMV, via disruption of virion assembly at the nucleocapsid. Case reports have shown that the use of leflunomide can lead to a decrease in the clearance of BK viremia [48,49,50], but the dose–effect has been poorly defined, and higher doses might be necessary to achieve BKPyV clearance [51], and higher doses are associated with higher prevalence of adverse effects, especially thrombotic microangiopathy [52]. On this basis, in 2010, a study suggested that inhibition of protein kinase inhibition with leflunomide is an effective therapy for BKPyV reactivation in addition to decreased IS [53]. Interestingly, Krisl et al. investigated the pharmacodynamics of leflunomide and found no association between A771726 (which is the active metabolite of leflunomide) and viral load reduction in BKPyV [54]. In 2023, Gole et al. treated all pediatric patients with BKPyV nephropathy with Leflunomide and IS reduction, and their experience showed rapid BKPyV clearance with the preservation of renal function [55]. Based on these contrasting findings and a recent meta-analysis, further RCT studies are needed to determine the role of Leflunomide in BKPyVAN, as it is not currently recommended for clinical use.

### 3.2. Cidofovir

Cidofovir is a nucleotide analog antiviral agent approved for the treatment of CMV retinitis associated with HIV infection that has been demonstrated to inhibit BKPyV replication in renal proximal tubule epithelial cells in vitro [56]. Because of the renal elimination of cidofovir and nephrotoxic effects, low-dose cidofovir (0.25–1 mg/kg) has been proposed as a therapeutic option for BKPyVN [57]. Evidence supporting the use of cidofovir for BKPyVAN is so far restricted to small case series and single-center studies [58,59]. In 2014, Kuten et al. conducted a study on 75 patients and used a mean of 13 doses of cidofovir in addition to reduction IS and showed that 71% of patients achieved BKPyV clearance at a median of 4.2 months. Patients who cleared BKPyV maintained stable graft function underwent no graft losses and had only a 15% decline in eGFR in the long term [60]. In 2023, Aksoy et al. conducted a retrospective study to assess head-to-head Leflunomide and cidofovir in 53 pediatric patients with BKPyVAN and persistent BKPyV viremia and showed similar results in terms of serum creatinine level and rate or graft loss efficacy for the treatment of BKPyVAN [61]. It is to be noted that a new agent, Brincidofovir, with an improved lipid formula of cidofovir, appears to be less nephrotoxic [62,63].

### 3.3. Fluoroquinolones

Levofloxacin is an inhibitor of the enzymes topoisomerase IV and topoisomerase II and has been demonstrated to inhibit BKPyV replication and cytopathic effects in a dose-dependent manner in a model using human proximal tubular epithelial cells [64,65]. Thus, several studies have been performed to assess the use of levofloxacin for prophylaxis of BKPyV-associated infection. Wojciechowski et al. showed that a 1-month course of levofloxacin resulted in a significant reduction in BKPyV viruria and viremia at 3 months post transplant compared to no prophylaxis [66]. Knoll et al. conducted an RCT study in 2014, with a 3-month course of levofloxacin initiated early following transplantation and was not able to show a significant difference in the occurrence of BKPyV viruria within the first year post transplant [67]. That same year, another prospective study showed that a one-month course of levofloxacin was not associated with a significant reduction in BK viremia at 3 months post treatment [68]. The results were confirmed in 2019 in a study in which no significant differences were observed in BKPyV viremia at 6 months between a 3-month course of levofloxacin and placebo in an RCT that included 200 patients [69]. Similarly, Lebreton et al. found no significant differences in BKPyV viremia or BKPyVAN outcomes with a 3-month course of levofloxacin [70]. It is worth noting that some case reports suggest a potential benefit of levofloxacin in treating BKPyV cystitis [71,72]. Although levofloxacin as prophylaxis does not appear to significantly prevent BK viremia and viremia post transplant, its role as a curative treatment of BKPyV infection still needs to be determined.

### 3.4. Intravenous Immunoglobulins

Intravenous immunoglobulins (IVIGs) are widely used as immunomodulatory agents but are also used as anti-infectious therapy as preparations may contain substantial amounts of neutralizing antibodies against bacteria and viruses. Commercial IVIG preparations contain potent neutralizing antibodies capable of neutralizing major BKPyV genotypes in vitro, and administration resulted in a significant increase in neutralizing antibody titers against BKPyV in vivo in patients treated with low (0.4 g/kg) or high (1 g/kg of body weight/day) IVIG doses [73,74]. Additionally, IVIG can suppress BKPyV replication and expression of the viral capsid protein 1 and large T-antigen on infected human kidney cells in vitro. IVIG reduced the number of virus-infected cells in a dose-dependent manner but did not affect viral release from infected cells [75]. Several monocentric experiences of the use of IVIG for pediatric and adult patients with BK viremia have shown significant viral load reduction in 60–86% of cases within 6 months in patients with concomitant decreased IS treatment [76,77,78]. Based on the neutralizing antibody titer level on the day of the transplant, a retrospective study showed that administration of IVIG during the first 3 months post transplant was associated with lower incidence of BKPyV viremia and BKPyVAN at 1-year post transplant [79]. Interestingly, IVIG (with IS reduction) was associated with the clearance of viral inclusion bodies and SV40 staining after treatment in patients with BKPyVAN in follow-up biopsies [80]. Another study confirmed that IVIG administration for 6 months significantly lowered BKPyV viral load in patients with persistent BKPyVAN. In this same study, no associations were identified between the response to IVIG and genotypes at FcγR3A (rs396991) and FcγR2A (rs1801274) single nucleotide polymorphisms [81]. IVIG was more effective in clearing viremia with faster and more complete resolution of viremia and clearing BK immunohistochemistry from tissue, compared to patients without IVIG. Patients with IVIG also tended to have fewer graft losses than patients without IVIG [82]. In 2023, an RCT by Rasaei et al. enrolling patients with BKPyV infection showed a significant decrease in BKPyV load 3 months after the administration of IVIG and leflunomide compared to IVIG alone. Therefore, the addition of leflunomide to IVIG appears to have improved efficacy in reducing BKPyV viral load. However, further studies with a larger sample size and longer follow-up and monitoring are required [83].

A comprehensive meta-analysis published in 2023 showed that the efficacy of IS reduction in combination with IVIG for serum BKPyV clearance was 87% (95% CI: 0.82–0.93; I2 = 45%), which was higher than for IS reduction in combination with leflunomide, cidofovir, or an mTOR inhibitor [52]. From an immunological standpoint, the use of IVIG could represent a more efficacious way of preventing the development of de novo DSA following IS reduction for BKPyV infection, as compared to mTOR inhibitors which are oppositely a risk factor for DSA development.

**Table 1:** Therapeutic armamentarium to treat BKPyVAN.

Treatment	Strategy	Sample Size	Follow Up	Outcome	Adverse Events	Ref.
Levofloxacin/ Ciprofloxacin	Preventive RCT: 3-month course of levofloxacin 500 mg per day vs. placebo, within 5 days of transplantation	154	46.5 weeks	No significant difference in the rate of BK viruria or viremia	Increased risk of resistant infection	[66]
	Therapeutic RCT: Patients with BK viremia were randomly assigned to receive levofloxacin, 500 mg daily, or placebo for 30 days	39	3 months	No significant difference in BK viral load reduction or allograft function	2 of the 22 patients in the levofloxacin group developed Achilles tendon pain	[67]
	Preventive RCT: 3-month course of ciprofloxacin vs. placebo at the time of transplant	200	12 months	Higher rates of BK and BKVAN at 12 months post transplant in the ciprofloxacin group	Higher rate of fluoroquinolone-resistant bacterial infections	[68]
	Preventive, non-randomized: 3-month course of ciprofloxacin compared to no ciprofloxacin, in patients who had undergone desensitization HLA- and/or ABO-incompatible transplantation	72	12 months	No significant difference between the rates of BK viruria, BK viremia, and BKVAN	Similar rate of bacterial infections	[69]
Cidofovir	Therapeutic, retrospective, uncontrolled: The effect of cidofovir with reduced immunosuppression in patients with high-level BK viremia or BKVAN	75	22 months	71% clearance of BKV with no loss of renal function and 15% decline in renal function 6 months after treatment	Leukopenia, anemia, and thrombocytopenia were frequent. Cidofovir was discontinued in one patient due to leukopenia	[59]
	Therapeutic, retrospective: Comparison of cidofovir and leflunomide	26	99 months	Similar serum creatinine levels and rates of graft loss between cidofovir and leflunomide	No adverse side effects associated with cidofovir and leflunomide	[60]
Leflunomide	Therapeutic, retrospective, uncontrolled: Effect of leflunomide (increasing dose) in patients with BKV infection	28	17 months	Patients required at least a 60 mg daily dose of leflunomide to achieve therapeutic levels and to clear BKV	Leukopenia, anemia, thrombocytopenia, and hepatotoxicity were common	[50]
	Therapeutic, retrospective: Comparison of leflunomide and no leflunomide	76	36 months	No difference in BKV clearance. Lack of correlation between A771726 concentrations and log change in BKV	Leukopenia, anemia, thrombocytopenia, and hepatotoxicity were common	[53]
	Therapeutic, retrospective, uncontrolled: Effect of leflunomide in patients with BKVAN after failure of other treatment approaches	55	50 months	Viremia decreased by 79 ± 37% and BKV clearance was observed in 76% of patients	Hematologic toxicity, bowel disorders, cholestasis, and rhabdomyolysis	[84]
	Therapeutic, retrospective, uncontrolled: Effect of leflunomide in pediatric patients	4	NA	All patients had undetectable levels of plasma BKV	No evidence of hematologic toxicity or hepatotoxicity	[54]
IVIG	Meta-analysis on the effect of different therapeutic interventions: Immunosuppression reduction alone or in combination with leflunomide, IVIG, cidofovir, or mTOR inhibitor (mTORi) therapy	986 patients in total, 117 treated with IS reduction + IVIG	NA	Compared to IS reduction alone, IS reduction + IVIG therapy offered a statistically significant benefit in serum BKPyV clearance	One case of deep-vein thrombosis	[51]
	Therapeutic, retrospective, uncontrolled: Effect of IVIG in patients with persistent BKV viremia and BKVAN	30	12 months	90% of patients cleared BKV viremia at 12 months	NA	[77]
	Preventive, retrospective: Early IVIG at transplant prevents BKV replication in patients with low NAb titers (high-risk group)	174	12 months	Reduced rate of BKV viremia at 12 months post transplant in the high-risk group treated with IVIG lower than that of the untreated high-risk group	NA	[78]
	Therapeutic, retrospective: Comparison of IVIG and no IVIG administration in patients with BKVAN	86	6 months	IVIG was associated with lower BKV DNA viral load	NA	[80]
	Therapeutic, retrospective: Comparison of IVIG and no IVIG administration in patients with BKVAN	50	60 months	The IVIG group more effectively cleared viremia, BKV immunohistochemistry, and resulted in faster resolution of viremia	NA	[81]

## 4. Ongoing Clinical Trials

Several interventional clinical trials are ongoing to evaluate the safety and tolerance of immune cell-based therapies against BKPyV including the use of CD8 cytotoxic T lymphocytes, virus-specific T cells, and ex vivo expanded rapidly generated virus-specific T cells (Table 2). These therapies based on T cell transfer are promising to specifically and directly target BKPyV but pose the challenge of reproducible cell manufacturing, the need for optimal HLA match between the donor and recipient, BKPyV-specific cell enrichment of preparations, and close monitoring of potential graft-versus-host disease (GVHD)-related side effects. Such studies are interventional phase I (NCT04293042, NCT05042076, and NCT05183490) and will closely evaluate the tolerance of infusion of BKPyV-specific T cells and the potential to decrease BKPyV load in the plasma of patients. Current clinical trials are also evaluating leflunomide plus an mTOR inhibitor (NCT04542733) and brincidofovir (NCT05511779), which appears to have less toxic renal side effects compared to cidofovir alone, in a randomized controlled manner. These trials will probably address the current limitations of the evaluation of these 2 drugs based on retrospective and uncontrolled studies. Building on promising results from previous small studies and their own findings [42,79], a French center is conducting a clinical trial (NCT04222023) to test the efficacy of IVIG in preventing BKPyV replication during the first year post kidney transplant. This study randomizes patients based on their BKPyV genotype-specific NAb titers at transplantation, with IVIG doses administered according to the BKPyV genotype (0.4 g/kg/day for genotype I and 1 g/kg/day for genotype II and IV). This study will also represent a proof-of-concept of the use of Nab titer to risk stratify patients at risk of BKPyV infection post transplant and for precision treatment based on biomarker assessment.

Finally, a promising strategy to prevent BKPyVAN in kidney transplant recipients is pre-transplant vaccination to induce high levels of neutralizing antibodies. Researchers at the National Cancer Institute developed virus-like particle (VLP) vaccines, modeled after HPV vaccines, which self-assemble and mimic native polyomavirus virions, eliciting robust and subtype-spanning immune responses in animal models. While these findings are encouraging, particularly the sustained antibody titers observed in mice and rhesus macaques, they require validation through human clinical trials [85].

**Table 2:** Overview of ongoing interventional clinical trials for the treatment of BKPyVAN.

Trial ID	Name	Country	Description	Phase	Treatment	Estimated Enrollment
NCT04293042	Treatment of BK Virus Infection With CTL Cells in Immunocompromised Transplant Patients (BK-CTLs)	USA	Open-label, single-arm clinical trial to assess the safety and efficacy of BK virus-specific cytotoxic T lymphocytes (CTL) in decreasing specific viral load in patients with BK virus viremia	1	Cytotoxic T lymphocytes	20
NCT05042076	BK With VST for Kidney Transplant Patients	USA	Open-label, single-group assignment study to assess the safety and tolerability of the transfer of BK-specific T cells. Safety will be assessed by determining the incidence and severity of acute infusion-related toxicities and the rejection of allograft acute GVHD. Efficacy will be determined by a decrease in BK viral load, BK clearance, and the resolution of clinical BK organ disease by week 12	1	Viral-specific T cells (VST)	20
NCT05183490	R-MVST Cells for Treatment of Viral Infections	USA	Open-label study to determine the safety and feasibility of administering ex vivo expanded rapidly generated virus-specific T (R-MVST) cells to patients with refractory viral reactivation and/or symptomatic disease-caused BK virus in solid organ transplant patients	1	Adoptive immunotherapy with R-MVST cells	36
NCT04542733	The efficacy of Everolimus With Reduced-dose Tacrolimus Versus Reduced-dose Tacrolimus in Treatment of BK Virus Infection in Kidney Transplantation Recipient (ELF)	Thailand	Open-label, randomized with parallel arms; the study will compare mTORi with reduced-dose tacrolimus and reduced-dose tacrolimus with or without leflunomide and assess change in plasma BK viral load at 3 months post randomization	NA	Reduced-dose tacrolimus + everolimus versus Reduced-dose tacrolimus + leflunomide	50
NCT05769582	Safety, Tolerability and Efficacy of AntiBKV as Treatment of BKV Infection in Kidney TransplantRecipients (SAFE KIDNEY II)	USA	Randomized double-blind and placebo-controlled study that assesses the efficacy of four doses of an investigational medicinal product (IMP) versus placebo in decreasing BKV DNAemia to undetectable levels at day 141 in kidney transplant recipients with BKV DNAemia	2/3	AntiBKV IMP versus placebo	180
NCT05511779	Study to Confirm of the Safety and Tolerability of Brincidofovir in Subjects With BK Virus Infection (Viremia) After Kidney Transplantation	Australia Japan	Open-label, randomized study that assesses the safety and efficacy of ascending dose of Brincidofovir versus standard of care (the use of the same immunosuppressant during prescreening) in change from baseline of BK viral load in plasma in kidney transplant recipients	2	Brincidofovir versus standard of care	36
NCT05325008	A Trial to Treat Polyomavirus Infections (BKPyV) in Kidney and Simultaneous Kidney Pancreas Transplant Recipients (BEAT-BK)	Australia	Randomized controlled study to evaluate immunosuppression reduction with or without IVIG in reaching composite ordinal outcome (all-cause death, allograft loss, eGFR decline, acute allograft rejection or BKV load > 1000 copies/mL, and immunosuppression load)	3	Immunosuppression reduction with and without IVIG	280
NCT04222023	Intravenous Immunoglobulins for Prevention of BKV Infection in Kidney Transplant Recipients According to BKV Genotype-specific Neutralizing Antibody Titers at the Day of Transplantation (BKANIG)	France	Randomized controlled study with recipients harboring a BKV Nab titer ≤ 4 log10 against the BKV genotype of their matched donor on the day of transplantation receiving (experimental group) or not (control group) IVIG treatment. The incidence of BKV viremia 6 months after transplantation will be evaluated	3	IVIG (0.4 g/kg/day for genotype I; 1 g/kg/day for genotype II and IV) versus no intervention	664

## 5. Conclusions

BKPyV-associated infection of the kidney transplant is recognized as a prime threat to prolonged graft survival. Performant tools are available for molecular diagnosis and have improved the characterization of the disease spectrum, going from urine viral detection to tissue evidence of BKPyV cytopathic effects in the kidney and severe graft functional alteration. However, future research efforts need to better assess interactions between the host and BKPyV, including casting light on the role of humoral and cellular immunity against BKPyV, developing more accurate markers of graft injury, and gaining a better understanding of the mechanism of antiviral drugs that may directly impact BKPyV replication. Additional perspectives of therapy include the use of neutralizing antibodies against BKPyV that would protect from viral replication and the design of a next-generation vaccine against BKPyV. These efforts will help foster the development of biomarker-based strategies for BKV monitoring, address the heterogeneity of BKV/host interactions, and tailor the management of immunosuppression and individual patient needs for adjunctive therapies.
